# Clinical Cases

**Published:** 1888-08

**Authors:** F. E. Stoaks


### CLINICAL CASES.

I. Mrs. W., aged sixty-five.—Has eczema on head and on neck behind the ears, where it began. Bald spots on top of head were covered with the eruption, extending into the hair, *matting it together* by clear, *watery*, and *sticky* discharge; of two years' standing. Considerable *itching* and *stinging*. *Graphites*$^{cm}$ (Swan), two doses, cured in three weeks and no return.

II. Mrs. G., aged thirty-seven.—Complained of *pain* and *sensitiveness in the coccyx*, especially when *sitting*. Was entirely relieved by *Petroleum*$^{cm}$ (Swan), one dose, in twenty-four hours.

III. Miss S., aged eleven.—Constipation, very obstinate for two years; stools *hard* and *dry;* desire for stool *delayed*, and causes considerable effort, and accompanied with *involuntary passing of urine. Alumina*$^{cm}$, four doses, cured.

IV. Mr. S., aged fifty-one.—Constipation from inactivity of rectum. Stools in *hard, round, black balls. Opium*$^{1m}$, one dose per week for three weeks, has nearly completed the cure. This case has baffled treatment of every variety for ten years. I am confident of curing it entirely.

V. Miss K., aged twenty-two.—Amenorrhœa, menses were substituted by leucorrhœa, which was *profuse* and *excoriating*, accompanied by *burning pain in right ovary*. Complexion pale and waxy, livid and yellow. Ars.$^{cm}$ (Swan), three doses, cured.

VI. Illustrating the quick action of high potencies in acute diseases. Mr. Wall, aged forty-five, called on me to relieve him of a colic located in *umbilical region*, which was only *relieved by pressure* and *bending double. Colocynthis*—a few pellets of 1M in water, a teaspoonful every time pain returned. He was entirely relieved after second dose. Time, thirty minutes. In these cases we have not much time to study our cases, but must "take in" the situation at a glance. So much for the value of knowing the characteristics. Could the hypodermic syringe do any better? I do not carry one or even have one in my office. Hence, I am free from the temptation of using it.

F. E. Stoaks, M. D.

